# Topics of Public Interest

**Published:** 1910-08

**Authors:** 


### TOPICS OF PUBLIC INTEREST

### Why New York Abrogated the Reciprocity Treaty with New Jersey

[ Editorial in *The Tribune*, July 15, 1910.]

NEW YORK AND NEW JERSEY DOCTORS.

The abrogation of reciprocity between New York and New Jersey in the matter of recognising licenses for the practice of medicine is much to be regretted and will be the cause, probably, of some inconvenience. Which of the two states will be the greater sufferer, in the persons of its physicians and patients, may be a matter of dispute; though we suppose that many more New York doctors will seek to practise in New Jersey than Jerseymen in New York; so that New York will probably suffer in the greater number of physicians and New Jersey in the greater number of patients who are denied their ministrations.

The number and size of the medical schools of New York and the lack of such institutions in New Jersey make a decided contrast between conditions in the two states. New York is one of the chief producers, perhaps the chief, of medical graduates, supplying them to every part of the Union, while New Jersey is almost entirely dependent upon institutions in other states for her supply. The present unfortunate controversy arises, however, over the alleged difference between the requirements of the laws of the two states in the matter of scholastic preparation for the practice and indeed for the study of medicine. New York requires three years of high school work as a preliminary to entering a medical school, the fourth year to be made up within the first two years of the medical course; while in New Jersey it is said to have been decided that a man without any high school training might enter a medical school and be duly graduated, provided he made up his high school work during the course. New York authorities, therefore, refuse longer to recognise New Jersey licenses on the ground of an inferior standard of qualifications and New Jersey now in return refuses to recognise New York licenses, denying that her standard has been lowered since the late reciprocal arrangement was made.

Apart from the technical merits of this specific case, the incident emphasises the great and growing desirability of the most uniform standards possible in professional qualifications, not only in these two states but throughout the nation. We have recently commented upon the beneficial influence which the Carnegie Foundation may have in promoting such uniformity. Perhaps this controversy will more or less directly conduce to that end.

---

**Where Shall Vacation be Spent—Typhoid Resorts—Bureau of Free Information at Albany Tells Where Not to Go.**

[*Brooklyn Times*]

Comparatively few persons, perhaps, know that the State of New York runs what might be called a summer resort bureau. If this knowledge was more common and put to the proper use, there would be fewer deaths from that dread malady, known as summer typhoid, which is usually brought on by a lack of proper sanitation. Every year toward the close for the summer season a large number of deaths occur from summer typhoid, and if one takes pains to look it up it will be found that many of the victims spent their vacation at the same resorts.

If you have not already made up your mind where to send your family this summer or where you are going yourself on your vacation, write the state commissioner of health at Albany a letter and he will be glad to tell you whether the places you have in mind are sanitary or not. In this way you can find out whether the lake, pond or river, near your hotel is polluted by sewage, if the plumbing and other appliances about the resort are proper and modern, and if there are any features, such as the disposal of garbage and refuse, the water supply and the general location which are objected to by the department. By doing this you may save yourself or some member of your family from a fatal sickness.

For several summers the state health commissioner, now Dr. Eugene H. Porter, has had agents going the rounds of the various summer resorts, inspecting conditions at the large hotels and boarding-houses. The principal matters which come under inspection are the disposal of sewage, the source of the water supply and the disposal of garbage. The agents report to the department head anything which they object to and a record of undesirable places is kept in the commissioner's office.

It is a violation of the law for hotels, boarding-houses and camps to discharge their sewage into lakes and streams, and if prosecuted the proprietors could be heavily fined in most cases. The commissioner, however, follows another course, which is regarded as much more effective in forcing the proprietors of summer resorts to comply with the law. Publicity is the punishment. The publication of the names and location of the unsanitary places would have a more salutary effect than a fine would.

Any proprietor of a summer resort who fails to comply with the law in regard to sanitation is liable to find printed in every paper in the state, under authority of the health commissioner, his name under a list of resorts which have not complied with the law. The fear of this, once the summer-resort owners realise it, quickly brings them to their senses. The proprietors are given a certain time to make changes recommended by the commissioner, and if they fail to comply with the law by the expiration of that time, they are placed on the blacklist.

Tens of thousands of persons go bathing every summer in lakes that are polluted by sewage, and expose themselves to disease and death. The state health commissioner has a list of every one of the hotels whose sewer system empties into the lake. If you want to enjoy your vacation and return healthier and happier than when you went away, get in touch with the state health commissioner and find out whether you are really going to a health resort or a place where your life and health may be endangered.

### Queer Medicines for Monarchs

The Marquise de Fontenoy, in *The Tribune,* writes the following bit of therapeutic antiquity, which will prove amusing if not interesting.

It seems difficult to realise that as late as a hundred years ago, that is to say, in 1810, some of the leading physicians in England, in attendance upon Queen Charlotte and holding office at court as her medical advisers, were prescribing extract of mummy, that is to say, drugs in which the powdered remains of the Egyptian Pharoahs constituted the chief ingredient. Half a century later—as recently as 1860—powders obtained through the crushing of mummies were still sold as medicine by the principal pharmaceutical chemists of Austria and Hungary.

In order to account for this employment of Egyptian mummies for the preparation of drugs it must be explained that great therapeutical virtues were ascribed to the ingredient employed by the ancient Egyptians in mummifying their rulers, their high priests and priestesses, and their queens. There was in particular a certain sort of asphalt which was considered unrivaled for its curative powers in cases of influensa, eczema, convulsions and epilepsy. In the eighteenth century, in fact until Napoleon I. virtually opened up the land of the Nile to scientific and archeological exploration, Egypt was more or less unknown and mummies were difficult to obtain. And thus one finds history recording Louis XIV. of France and Empress Catherine the Great as receiving as highly prized gifts gold and silver boxes containing either entire mummies or else fragments thereof.

Nowadays so many of the ancient Acropoli of Egypt and of the Soudan have been discovered and opened up that the crop of ancient mummies has become almost larger than the demand. They have become, in a different and more metaphorical sense, a veritable drug on the market, and not long ago were being shipped to this country in quantities for use in the fertilisation of the onion beds of New Jersey. While this is, of course, calculated to offend one's sense of propriety and of the respect that is due to the departed, even if they have been dead for four thousand or five thousand years, still it is, after all, less repellent than the almost cannibalistic idea of modern kings and queens taking their brother monarchs of Mosaical times internally as medicine.

